# Applying movement ecology models to comparative cognition experiments: a field test in hummingbirds

**DOI:** 10.1098/rspb.2025.0717

**Published:** 2025-07-23

**Authors:** David J. Pritchard, T. Andrew Hurly, Theoni Photopoulou, Susan D. Healy

**Affiliations:** ^1^ School of Psychology, Newcastle University, Newcastle upon Tyne, UK; ^2^ Department of Biological Sciences, University of Lethbridge, Lethbridge, Alberta, Canada; ^3^ School of Biology, University of St Andrews, St Andrews, UK; ^4^ Centre for Research into Ecological and Environmental Modelling, University of St Andrews, St Andrews, UK; ^5^ Centre for Statistics in Ecology, Environment and Conservation, Department of Statistical Sciences, University of Cape Town, Rondebosch 7701, Western Cape, South Africa

**Keywords:** landmarks, navigation, spatial cognition, spatial learning, memory, movement ecology

## Abstract

Traditionally, evidence for spatial learning in animals involves experiments; however, tools developed for analysing tracking data are increasingly being used to infer the use of spatial memory and other cognitive processes in animal movements. In this study, we combined these statistical models with field experiments to analyse how patterns of hummingbird movements change as birds learn a rewarded location. Using hidden Markov models (HMMs), we used both changes in two movement states as well as experimental behavioural measures of spatial memory to determine how searching performance and behaviour changed as birds gained experience and how important local landmarks were for guiding search. Regardless of whether birds had a single training trial to learn a flower’s location or 12 repeated trials, hummingbirds learned the location of the flower although performance suffered when we removed the local landmarks. While the hovering locations suggested that removing landmarks led to a slight decrease in accuracy compared with when landmarks were present, the HMMs suggest that this was part of a larger shift from a memory-led search strategy to a more systematic searching process. Our results suggests that models used in movement ecology could provide a valuable tool for experiments in comparative cognition.

## 1. Introduction

Spatial memory is crucial for efficient foraging. The ability to learn and return to locations enables mobile animals to return to profitable foraging locations, avoid locations they have already exploited and travel efficiently to and from different sites [1]. This strong association between spatial memory and foraging often features in two research fields: experimental animal cognition and movement ecology. Experimental tests of spatial memory are often framed as foraging tasks, such as recovering caches [2] or collecting food in a maze [3], while ecologists studying foraging movements are becoming increasingly interested in how such movements indicate the acquisition and expression of spatial memory [4,5]. Despite this common interest, however, differences in the methods that researchers use, the data they collect and even the scale over which studies take place, mean that knowledge about the mechanisms of spatial cognition originating in comparative cognition is not straightforward to apply to the observations of animal movements in the wild studied by movement ecologists.

Movement ecologists have mostly attempted to look for signatures of spatial memory in observed patterns of movement over multiple kilometres. These signatures could include bias in animal movements to previously visited locations or routes, greater efficiency in paths over time or attraction of animals to locations that seem to fall beyond the range of their senses [4–7]. Identifying the presence of memory in movement data is complicated, however, as it is often difficult or impossible to rule out alternative explanations, such as animals directly sensing some cue that attracts or repels them and biases their movement accordingly [6]. The same complications also make attributing memory to any specific remembered information in the environment very challenging, and so most studies have been focused solely on *if* memory is being used rather than the information content of that memory.

In contrast, studies of animal cognition explicitly focus on the content of spatial memory and how animals use the information they perceive and remember to estimate their location and plan their future movements. These experiments often occur on a much smaller scale than movement ecology studies but have revealed a wide range of different cues animals can learn and use to navigate, including local landmarks and distant panoramas, larger scale maps based on magnetism or odour and directional information from magnetic and celestial compasses [7]. Unlike movement ecology studies of wild animals, in which it is often difficult to say if learning has occurred, cognition researchers explicitly train animals to learn about a location and then test what the animals have learned using experimental manipulations. Such experimental tests could, therefore, complement research in movement ecology, providing insights into how animals using memory behave and what cues they might use. But bringing these two disciplines together raises challenges. For example, the scales studied by movement ecologists are typically much larger than is usually logistically feasible for traditional spatial memory experiments.

What is measured and analysed in cognitive experiments and movement ecology studies also differs. Researchers working on cognition traditionally quantify the effect of spatial memory by recording specific discrete behaviours, such as where an animal digs or pecks for food [8] rather than analysing the continuous searching movements studied by movement ecologists [4]. This makes comparing the two approaches or applying laboratory insights to the wild, potentially challenging as laboratory researchers almost never collect the kind of data analysed by movement ecologists. Even if there were movement data from laboratory experiments to analyse, differences in training and tasks would still make straightforward comparisons between laboratory studies and wild animal movements difficult. For example, animals in open-field spatial memory experiments, such as hidden-food paradigms [8,9], are often trained over many trials to be as certain about a rewarded location as possible. This reduces, and is intended to reduce, the variation in performance within individuals to its minimum to gain the best estimate for where the animal ‘thinks’ the goal should be and so determine the precision and accuracy of their spatial representation and how it is affected by experimental manipulations. But as a side effect, it means that the behaviours seen in the laboratory during spatial memory tests may not represent well how wild animals actually move and search when using memory. Animals who have learned about their natural environments through exploration and experience will probably not be as certain about a remembered location or have access to such reliable information, as do highly trained animals in a dedicated experimental environment. Understanding how the mechanisms examined in comparative cognition experiments relate to movements at the scale studied in movement ecology, therefore, requires more than simply taking the laboratory to the field or comparing wild animals with those trained in captivity. It requires a broad understanding of how memory can influence different aspects of behaviour, from continuous movement to discrete decisions, as well as how these effects of memory are affected by the experience an animal receives and its confidence in its own knowledge.

In this experiment, we set out to build such a bridge between movement ecology and animal cognition by applying approaches or tools developed from each to test whether combining the approaches might offer a unique view on how memory shapes searching. We, thus, trained free-living hummingbirds to expect a reward in a fixed location, controlled the experience and confidence of the birds, and manipulated the availability of local landmarks (all traditional cognitive test methods). But unlike traditional cognitive tests, we did not restrict our analysis to discrete behaviours but also tracked the three-dimensional flight paths of birds. We then applied hidden Markov models (HMMs), commonly used in movement ecology [10], to identify distinct movement states in the three-dimensional flight path and to analyse how the likelihood of being in these states, and switching between states, changes depending on the bird’s location and the presence or absence of familiar local landmarks.

## 2. Methods

### (a) Subjects and experimental site

The subjects in this experiment were 14 male rufous hummingbirds *Selasphorus rufus*. Each bird was individually identifiable by a coloured ink mark applied to its breast and occupied a territory around one of 25 artificial feeders suspended 3 m from the ground and positioned along the Westcastle valley of the Eastern Range of the Canadian Rockies in Alberta, Canada (49°29*′* N, 114°25*′* W). This experiment was conducted between May and July 2014 when the males had migrated to Canada from Mexico for the breeding season.

### (b) Pretraining

**Figure 1.**
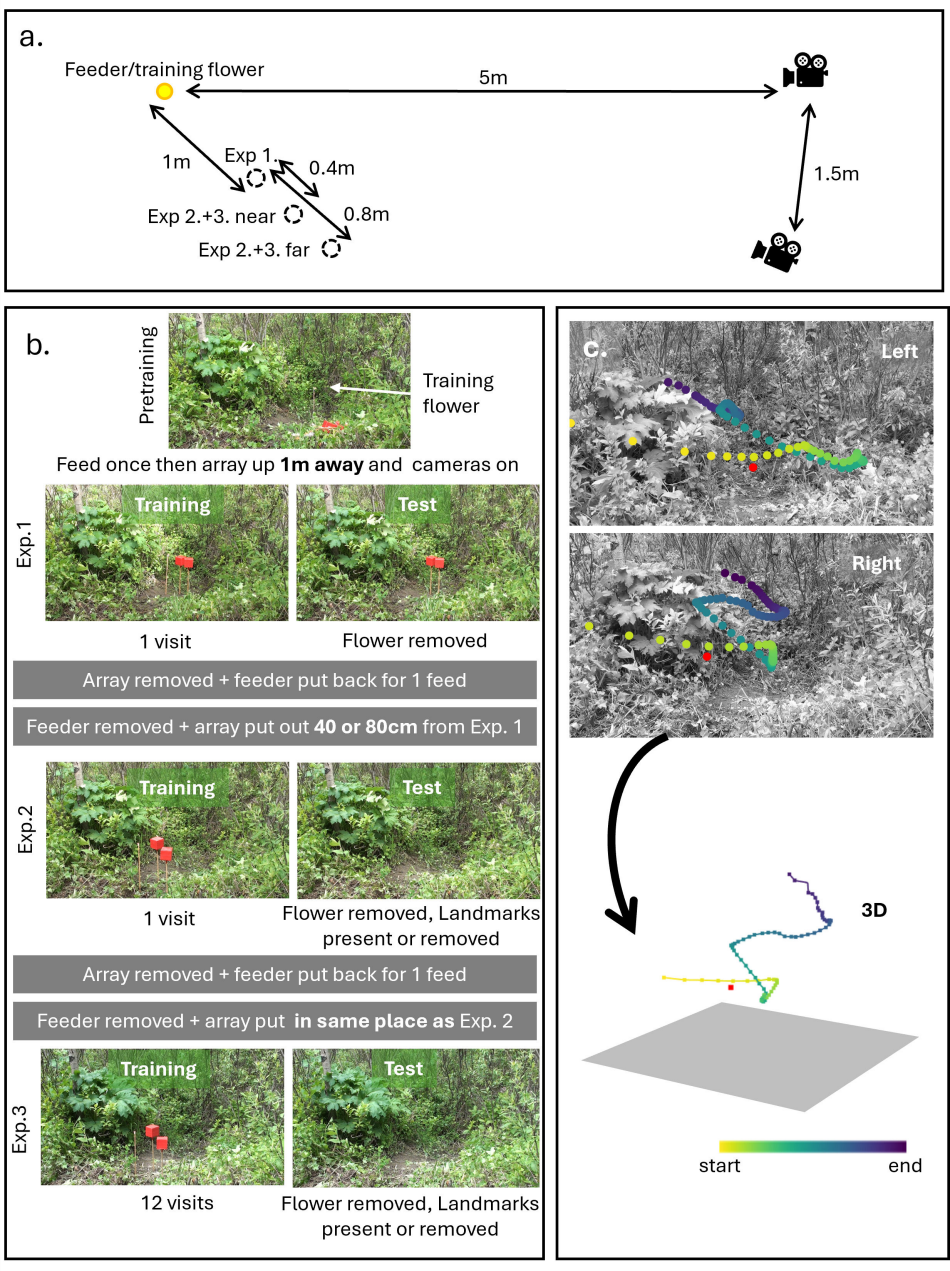
Experimental design used in this study. (a) Layout of the training flower used in training, the positions of the test flower in experiments 1−3 and the cameras. (b) Experimental design with illustrations from the video of a bird in the landmarks removed group. (c) Flight paths were quantified by hand from each camera and then converted to three dimensions using stereo calibration.

Prior to starting the experiment, hummingbirds were trained to find 25% sucrose (w/w determined using a refractometer) from a yellow ‘training flower’, 6 cm in diameter, on the feeder. On the day each bird was to be tested, we removed the feeder and placed the training flower on a 62 cm stake directly beneath the feeder’s location. We then positioned two Sony Handicam camcorders on tripods 1.5 m apart and 5 m from the training flower (figure 1a). The views from both cameras were centred on the location of the training flower. After the resident bird fed from the training flower, we started the cameras, removed the training flower and set up the experimental array for the first experiment (figure 1b).

### (c) Experiment 1: single experience, landmarks present

The experimental array consisted of a novel pink ‘test flower’, 3 cm in diameter at the top of a 62 cm long wooden pole, with two landmarks 30 cm away placed with one to the northwest and one the northeast of the flower in an equilateral triangle. The landmarks were red cubes make of card and red duct tape, 10 × 10 × 10 cm, on top of wooden poles 62 cm long (figure 1b). In Experiment 1, the test flower was always placed 1 m from the previous position of the yellow training flower (figure 1a). We then synchronized and calibrated the cameras, using the method described in the section below. During the first experiment, we removed the feeder from the hummingbird’s territory and allowed the territorial bird to feed once from the test flower in the experimental array. If the territorial bird did not feed from the test flower after multiple attempts, as was the case at three sites, we turned off the cameras, removed the test flower and allowed the hummingbird to feed 2−4 more times from the training flower at different locations more than 2 m from the location of the experimental array. We then started the test again from the beginning with the training flower below the feeder. If, as at two sites, the birds continued to avoid the test flower, we trained the birds to visit the test flower. We did this by allowing the birds to feed from the training flower at a location more than 2 m from the location of the experimental array. When the bird had fed once, we placed the pink disc of the test flower over the larger yellow disc of the training flower to make a hybrid flower, which we then allowed the birds to feed from once. After the bird had fed from the hybrid flower, we removed the yellow disc so that only the test flower remained. After the bird had fed from the test flower, we moved the flower to a new location, again more than 2 m from the location of the experimental array and allowed the bird to feed again. Having fed from the test flower no more than four times, the flower was removed, the feeder replaced and the test started again, from the beginning on the following day. We did not see any specific effect of this extra training on later performance (electronic supplementary material, figures S5–S6 and table S3). After the bird had fed from the test flower and flown away, we removed the test flower but kept the landmarks in place. We synchronized the cameras once more, before retreating to wait for the bird to return. Following the second visit by the bird to the experimental array (now with the flower removed), we synchronized the cameras one last time, removed the landmarks and replaced the feeder. Both visits were recorded by the cameras. After the bird had fed once from the feeder, we removed the feeder again and replaced the experimental array for the second experiment.

### (d) Experiment 2: single experience, near or far, landmarks present or absent

The experimental array was moved between Experiments 1 and 2. For birds in the Near condition, we placed the test flower 40 cm from the location of the test flower in the test in Experiment 1. For birds in the Far condition, we placed the test flower 80 cm from the location of the test flower in the first test (figure 1a). As before, we placed the landmarks 30 cm from the current location of the test flower, to the north in an equilateral triangle with the flower. We then started recording, calibrated and synchronized the cameras. Once the territorial bird had fed once from the flower, we synchronized the cameras, removed the test flower, and, for birds in the ‘No Landmarks’ group, we also removed the landmarks (figure 1b). For birds in the ‘Landmarks’ group, the landmarks remained in place. After the bird had visited a second time and flown away, we synchronized the cameras and stopped recording, removed the landmarks if they were present and replaced the feeder. As in Experiment 1, all visits were recorded by the cameras. Once the territorial bird had fed once from the feeder, we once again removed the feeder and returned the experimental array to the same location for Experiment 3.

### (e) Experiment 3: multiple experiences, landmarks present or absent

For the third experiment, we allowed the hummingbird to feed 12 more times from the test flower, a number based on previous studies of hummingbirds under similar conditions [11]. Once the bird had fed and flown away after the 12th visit, we synchronized the cameras and removed the test flower. Following the 11th visit, we started recording, calibrated and synchronized the cameras and allowed the bird to feed for a 12th time from the test flower. As in Experiment 2, for birds in the ‘No Landmarks’ group, we removed the landmarks alongside the flower, while for birds in the ‘Landmarks’ group, the landmarks remained in place (figure 1b). After the bird had visited once more, we synchronized the cameras for a final time, we removed the landmarks if they were still out, stopped recording and replaced the feeder. Due to battery constraints, in Experiment 3 we recorded only the 12th visit and the test itself.

### (f) Synchronization, calibration and triangulation

To synchronize the two cameras, we hit a red screwdriver against a white mallet in view of both cameras. We later edited the collected videos in Sony Vegas, matching up the frames in which the screwdriver struck the hammer on the left and right videos and splitting the videos into three distinct sections: calibration, training (the first visit, with the flower present) and testing (the second visit, with the flower removed). Having saved the new, synchronized video segments, we then calibrated the cameras and extracted the flight paths of the birds.

To perform the stereo calibration, we first used ffmpeg (ffmpeg.org) to extract the frames from each of the calibration videos, then used a custom MATLAB script to identify frames where the entire chequerboard was clearly visible and had been from three frames before to three frames after. Having identified good images of the chequerboard from both cameras, we used the Stereo Camera Calibration App in MATLAB to calibrate the cameras based on the view of the chequerboard from the left and right cameras in the chosen images. Because this method required the chequerboard to be rectangular, with an odd number of squares on one axis and an even number of squares on the other, one row of black squares was removed from the calibration images using a white brush in Microsoft Paint prior to calibration. The known dimensions of the chequerboard resulted in the absolute distances between the cameras, and by extension objects reconstructed after calibration, being accurate to the distance in real life.

For each training and testing video segment, we extracted the frames from the videos, which were sorted depending on what the bird was doing. In training videos, the frames were split into ‘In’ (from the first view of the bird to feeding from the flower), ‘Feeding’ and ‘Out’ (from leaving the flower to disappearing from view). In test videos, we kept the entire period from the bird entering the shot, to the bird leaving. Using a custom MATLAB script, these images were converted to .avi video files and imported into Kinovea (kinovea.org) to extract the trajectory of the birds from each of these videos by clicking on the head of the bird in each frame. When the bird was moving too fast to be seen clearly, we instead clicked on the leading edge of the blur seen as the bird moved. Overall, we collected six sets of trajectories for each test at each site, the head coordinates during the flight in as seen from each camera, the head coordinates during the flight out as seen from each camera and the head coordinates during the flight in the test phase as seen from each camera. This resulted in a dataset of 2679 locations from 14 birds in Experiment 1, 1830 locations from 14 birds in Experiment 2 and 1748 locations from 14 birds in Experiment 3.

Once we had both the stereo calibration data from the cameras and the *x*, *y* coordinates of the bird’s head from the videos, we were then able to reconstruct the *x*, *y* and *z* coordinates of the head of the bird in relation to the camera by using the triangulate function in the MATLAB stereo calibration package.

### (g) Data analysis

During the test, we recorded the flight of the bird from a pair of stereo-calibrated cameras and extracted the three-dimensional position of the bird at each time point (electronic supplementary material, figure 1c). We analysed the three-dimensional paths using two approaches: (i) to examine typical spatial learning and memory measures, we recorded where birds stopped and hovered in place (‘stops’), identified as periods in which the step length between video frames was less than 1 cm and how close birds flew to the flower’s location (‘fly-bys’) following the previous study examining spatial memory and hummingbird flight paths [11]. Data about stops and fly-bys are reported as means ± standard error (s.e.); (ii) to identify probable movement states, we use HMMs to analyse the birds’ flight paths in their entirety and how these changed with distance to the flower’s previous location.

#### (i) Analysis of stops and distances

To determine how our treatments affected where birds hovered and how close they flew to the flower’s location, we used generalized linear mixed models (GLMMs) to compare performance in adjacent experiments (1 versus 2 and 2 versus 3) as these models can compare both within-subject (differences between experiments) and between-subject effects (differences between landmark groups). We ran these models using the lme4 (v. 1.1−35.3) package in R [12]. The response variable was standardized, scaled and a constant added to avoid negative values. The explanatory variables were Experiment (1 versus 2 or 2 versus 3) and Landmark group (present versus absent). For analyses comparing Experiments 1 and 2, we also initially included the distance the flower moved between experiments (Near versus Far); however, as models with this variable were never selected, we removed this variable to restrict the possible models being evaluated. We fitted all possible combinations of explanatory variables using the dredge function in MuMIn (v. 1.47.5) [13], which compares models based on Akaike information criterion with correction for small sample sizes (AICc), delta weight and Akaike weight. In cases in which the delta weight of between models was less than 2 [14], model outputs were averaged using the ‘model.avg’ function in the MuMIn package. While results from individual GLMMs are reported with the *t* coefficient, the results from averaged models are reported with the *z* coefficient, which represents the average of the coefficients in the constituent models. Results from model selection, including the weights used in averaged models, can be found in the electronic supplementary material, table S2.

#### (ii) Analysis of movement states

We used multivariate HMM to analyse the reconstructed trajectories of the birds under each experimental set-up. HMMs are models for analysing time series of observations recorded at regular time intervals. They are now commonly used in the animal movement context for inferring the underlying movement modes that give rise to observed movement metrics (e.g. [15]). The classical HMM formulation assumes a first-order dependence between the underlying states and between the observations and the underlying states. HMMs for animal movement often involve modelling two derived variables (one scalar and one angular) from relocation data, e.g. the step length and turning angle between consecutive observed locations in the case of terrestrial movement. Flying animals move in a volume rather than on a plane, and this can easily be accommodated in HMMs by including an extra angular component, or data stream, as we do here. The trajectory reconstruction provided a three-dimensional step length, a yaw angle (nose side to side) and a pitch angle (nose up and down). We modelled step length using a gamma distribution, which is suitable for modelling continuous, positive-valued variables, as in step length (electronic supplementary material, figure S1). There were some steps which had zero length (no displacement), due to birds stopping and hovering, and we accounted for this by estimating an additional parameter for the probability mass of the observations that are zero. We modelled both angular state variables using a wrapped Cauchy distribution, which has two parameters, the mean and concentration, where the latter relates to how peaked the distribution is (electronic supplementary material, figure S1). We fitted models where either both parameters were estimated or only the concentration parameter while assuming the mean to be zero.

#### Model structure and implementation

An unobserved Markov chain was assumed to determine the movement states and the parameters of the state-dependent distributions associated with the observed movement variables. Our research question pertains to inferring information about the different types of movement behaviour carried out by rufous hummingbirds. We fit the model to all birds jointly and therefore estimate one set of parameters across individuals. The HMM parameters are estimated using numerical maximization of the likelihood, implemented in R [16], using the momentuHMM package [17]. In this package, the computation of the covariate-dependent transition probability matrices and the forward algorithm is coded in C++. The forward algorithm is an efficient way of evaluating the likelihood and is one reason for the popularity of HMMs—it makes them fast to fit. It corresponds to a recursive calculation of the likelihood with computational costs only linear in the number of observed time points and renders numerical maximum likelihood estimation feasible [18]. We only fit models with two states, based on biological knowledge of the movement behaviour of the birds during the experiment, and guided by the aim of the analysis (electronic supplementary material, figure S1). We expected birds to exhibit more rapid, directed movement towards an area of interest, followed by more sinuous and perhaps slower movement while searching for a flower to feed from. We did not consider models with different numbers of states, following the pragmatic approach outlined in [19].

#### Likelihood of the hidden Markov models

The likelihood of an HMM with N states and observation vectors $z_{1}$, ..., $z_{T}$ can be written as a matrix product,


(2.1) $$L=\delta P\left(z_{1}\right)\prod_{t=2}^{T}\Gamma_{t}P\left(z_{t}\right)1^{\prime },$$

where δ is a row-vector containing the initial state distribution, $\Gamma_{t}$ represents the N × N transition probability matrix at time point *t* and 1 is a row-vector of ones. **P** denotes a N × N diagonal matrix containing the values of the N joint state-dependent densities evaluated at the observation vector ${\text{z}}_{t}$. We assume the observed variables to be contemporaneously conditionally independent, given the current state. Thus, for each state, the joint state-dependent density is the product of the univariate state-dependent densities that are associated with the observed variables. In this analysis, ${\text{z}}_{t}$ corresponds to the vector: step length, yaw angle (side-to-side movement) and pitch angle (up-down movement) observed at time *t* along the movement track. We model the three state variables (step, yaw and pitch) with distance between the bird and the test flower (referred to as current distance to flower from here on) as a covariate on the probability of transitioning between states. We also included the presence of landmarks as a covariate in Experiments 2 and 3—landmarks were present for all birds in Experiment 1. We implemented this in two ways, (i) we included the presence of landmarks as a covariate on the state-dependent distributions, to test whether the parameters of the estimated component distributions of movement states change when landmarks are present and (ii) we included the presence of landmarks as a covariate on the transition probabilities, the same as with current distance to flower. To investigate the influence of distance between the bird and the previous location of the flower (m), and the effect of the presence of landmarks (0/1) on movement behaviour, these two covariates are incorporated into the model either on the transition probabilities (independently or as interacting covariates) (equation (2.2)) or on the mean of the state-dependent distribution for step length (equation (2.3).

For a model with distance from flower and the presence of landmarks interacting to affect the transition probabilities, the linear predictor would be


(2.2) $$\ln\;\left(\frac{\gamma_{ij}(t)}{\gamma_{ii}(t)}\right)=\beta_{0ij}+\beta_{1ij}{\text{Distance}}_{t}+\beta_{2ij}\text{Landmarks}+\beta_{3ij}{\text{Distance}}_{t}\text{Landmarks},$$

where $\gamma_{ij}(t)$ denotes the probability of switching from state *i* to state *j* at time *t*. The effect of absence of landmarks is absorbed into the intercept term. For a model with the presence of landmarks acting on the mean of the state-dependent distribution for step length, the linear predictor would be


(2.3) $$\ln\;\left(\mu_{\text{step }}\right)=\beta_{0ij}+\beta_{1ij}\text{Landmarks},$$

where, again, the absence of landmarks is absorbed into the intercept, and the slope coefficient corresponds to the presence of landmarks. Given we are not fitting any random effects, the log-likelihood of interest is the sum of log-likelihoods corresponding to the different birds within each experiment.

#### Model assessment

We used the AIC weights to choose between models with different covariates for each of the three experiments (electronic supplementary material, table S1), but all models had two states. For the best model from each experiment, we used the Viterbi algorithm to obtain the most likely state sequence and we also calculated the state probabilities, which give the probability of being in a given state at each point in the observed time series. We examined model fit for each experiment by calculating the pseudo-residuals for each of the state-dependent variables (step length, yaw and pitch) and checking their distributions and the residual autocorrelation, using the acf function in R [16] (electronic supplementary material, “Diagnostics on best models”).

## 3. Results

### (a) Experiment 1: does spatial memory shape search after a single visit?

**Figure 2.**
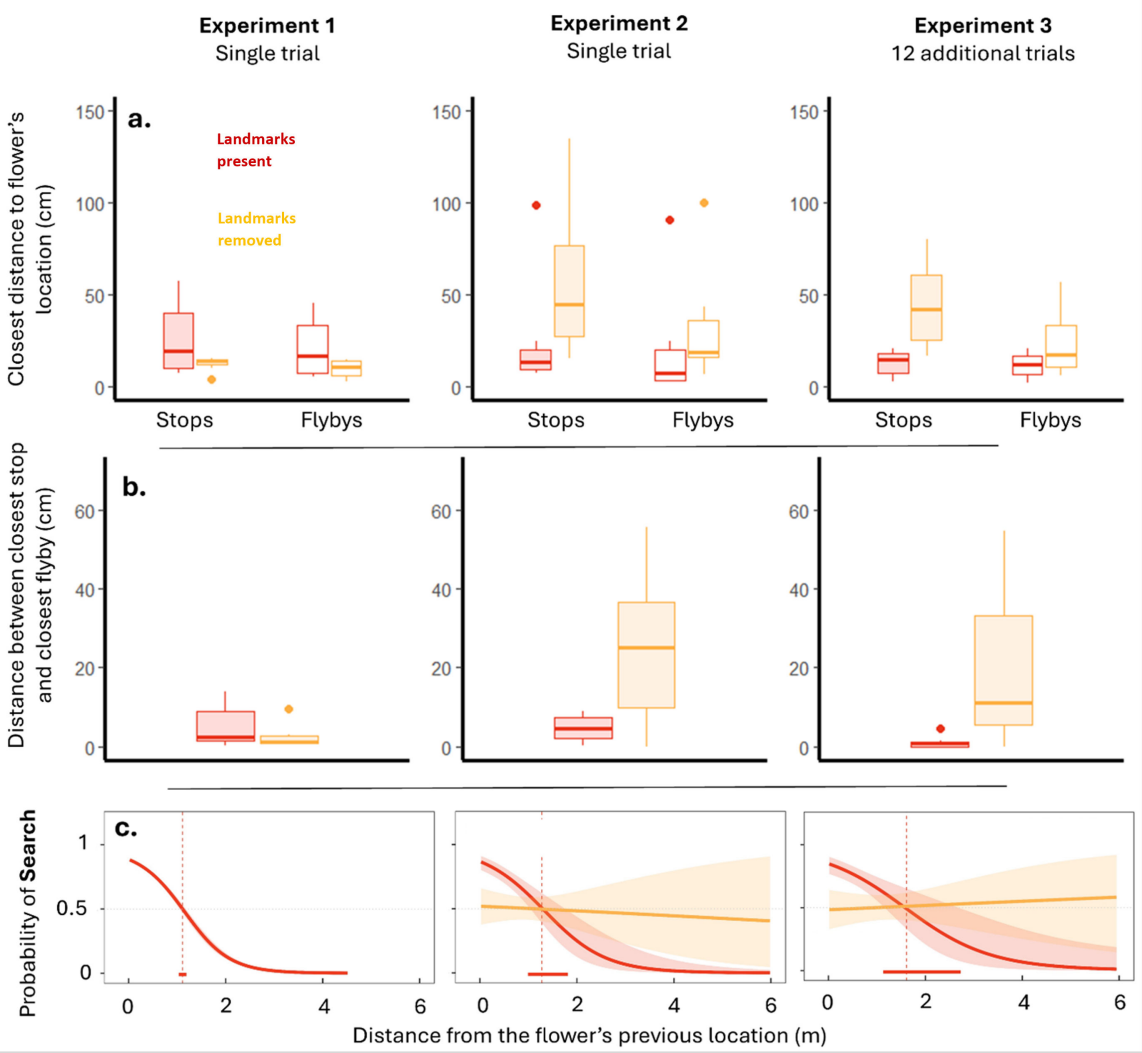
Removing local landmarks impacts searching precision and structure. (a) The closest distance that birds stopped (filled) or flew (open) to the flower’s location when the landmarks were present (red) or removed (yellow). In Experiment 1, all birds had the landmarks present and so colour in 1 a reflects the groups to which birds were assigned in Experiments 2 and 3. (b) The difference between closest stop and closest fly-by when landmarks present (red) or removed (yellow). Again, birds in Experiment 1 always had the landmarks present and so colour in one dimension reflects the groups to which birds were assigned in Experiments 2 and 3. (c) HMM results displaying how stationary state probability changed with distance to the flower’s previous location and whether the landmarks are present (red line) or removed (yellow line). The dashed line and bar along the *x*-axis display the distance (± 95% confidence interval) where birds crossed over 50% probability of being in Search.

When the flower was removed, all birds flew close and stopped close to the flower’s previous location. We recorded both the closest distance between the flight path and the flower’s location (closest ‘fly-by’), and the distance of the closest stop to the flower’s location. Birds often flew slightly closer to the flower’s location than the distance of the closest stop (closest fly-by: 15.07 ± 3.4 cm; closest stop: 19.03 ± 4.1 cm; figure 2a), but the difference between these two measures was only a few centimetres (figure 2b). This suggests that the birds stopped when they were almost at their closest to the flower’s location.

Note that for the purposes of illustration in the figures, we have separated the birds in Experiment 1 into two groups, even though all birds had the same experience in this experiment. This is because when analysing the data, we saw that the birds in Experiment 2 that had landmarks removed had tended to fly and stop closer to the flower’s former location in Experiment 1 than had the birds that in Experiment 2 did not have the landmarks removed. Birds were assigned to ‘landmarks present’ and ‘landmarks removed’ groups before any data were collected in Experiment 1, meaning that these differences were not due to their assigned group, but probably represent pre-existing differences between the individual hummingbirds that made up each group. Although the difference between these two groups was not significant in Experiment 1, it did impact on the comparisons between Experiments 1 and 2 in a way that would be obscured if the birds in Experiment 1 were pooled together in the figures.

**Figure 3.**
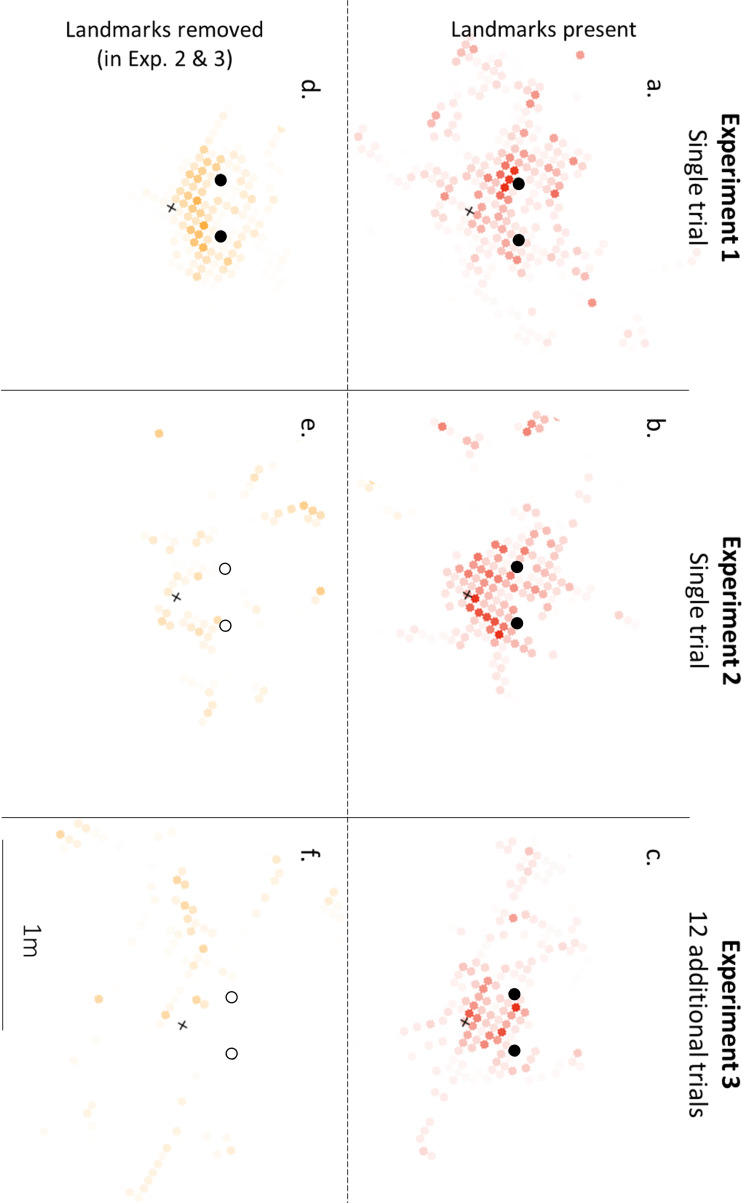
Heat maps of the location of datapoints assigned to the Search state by the Viterbi algorithm for the most successful model. Space was divided into 5 cm diameter squares, the black dots are the landmarks and the flower’s location is represented as a +. Darker points mean that more birds were in the Search state in that square.

This ‘tuning’ of behaviour around the flower’s location was also seen in the HMM results. The models identified two states: a faster and more direct style of movement, hereafter referred to as ‘Travel’, and a slower, more sinuous style of movements we refer to as ‘Search’. In the best fitting model, the probability of switching between these states was determined by the bird’s current distance to the flower’s previous location (electronic supplementary material, table S1). The closer the hummingbird was to the flower’s former location, the more likely it was to switch from Travel to Search (electronic supplementary material, figure S2), resulting in the probability of being in the Search state increasing with decreasing distance to the flower’s location (figure 2c). To examine where the Search state occurred around the landmarks, we generated heat maps by overlaying plots from each individual bird, to show where the Search state was concentrated. Peaks in the Search state were observed around the rewarded location, particularly in the space between the flower’s former position and the landmarks (figure 3a,d).

### (b) Experiment 2: effects of removing landmarks on the use of spatial memory after a single visit

In Experiment 2, the birds with the landmarks behaved just as they had in Experiment 1 (closest stop: 25.3 ± 12.4 cm; closest fly-by: 20.7 ± 12.02 cm; figure 2a). But the birds without the landmarks in Experiment 2 stopped further from the flower’s location (57.2 ± 16.5 cm), and their flight path never got as close to the flower’s location as it had in Experiment 1 (32.4 ± 11.9 cm; experiment × landmark interactions: GLMM for closest stop: −*t* = 3.97, *p* < 0.0001; averaged model for closest fly-by: *z* = 2.54, *p* = 0.011; figure 2a). Stopping accuracy was particularly affected by the removal of the landmarks. When the landmarks were present, the closest stop and the closest fly-by were at similar distances to the flower’s previous location, differing only in a few centimetres (difference between closest stop and closest fly-by: 4.6 ± 1.3 cm). When the landmarks were removed, birds’ stops became significantly less accurate than fly-bys, with the closest stop now tens of centimetres further from the flower’s location than from the closest fly-by (24.8 ± 7.4 cm; difference between closest stop and closest flown: experiment × landmark interaction: GLMM, *t* = 3.84, *p* = 0.00012, figure 2b). As mentioned in the methods, we did not see any effect of the distance moved between experiments, with models including this as a variable never being selected or included in any averaged final model.

In addition to stopping further from the flower’s previous location, we also found that birds with the landmarks removed also significantly increased the travel distances between stops (mean ± s.e. change in distance between stops in Experiments 1 and 2: Landmarks: increase of 7.4 ± 8.6 cm; No Landmarks: increase of 42 ± 35.4 cm; distance between stops: experiment × landmark interaction: GLMM, *t* = 5.16, *p* < 0.0001). This effect was, however, quite small and largely driven by one bird that stopped in two locations almost 3 m apart (1 distance greater than 2.5 m of 128 total). When this result was removed, there was no effect, suggesting that birds did not space their stops because the landmarks had been removed.

Removing the landmarks did, however, influence where the HMM-identified states occurred. As in Experiment 1, the models identified states corresponding to Travel (faster with little turning) and Search (slower with increased turning). In the best fitting model, the probability of switching between these states was determined by an interaction between the bird’s current distance to the flower’s previous location and whether or not the landmarks were present. When the landmarks were present, the birds behaved very similarly to how they behaved in Experiment 1. The closer the birds were to the flower’s previous location, the greater the likelihood of switching from Travel to Search (electronic supplementary material, figure S2), and so there was a greater likelihood of being in the Search state around the flower’s former location (figure 2c). When the landmarks were removed, however, the probability of switching state (electronic supplementary material, figure S2) or being in the Search state was the same at all distances from the flower’s location, with birds no more likely to be Searching or Travelling along the entire flight path (figure 2c). This effect of removing the landmarks can also be seen in the heatmaps of where Search occurred around the rewarded location. Birds with the landmarks had a similar focused pattern of searching as they had in Experiment 1, with movements assigned to Search tightly focused around the rewarded location or between the flower’s former location and the landmarks (figure 3b). The birds without the landmarks, however, had a much more diffuse distribution of movements assigned to Search, without any apparent focus on the rewarded location (figure 3e).

### (c) Experiment 3: effects of additional experience on reliance on landmark memory in search

There was some evidence that birds with the landmarks in Experiment 3 continued to stop (12.5 ± 2.6 cm, averaged model for closest stop: landmark: *z* = 2.051, *p* = 0.04, figure 2a) closer to the flower’s location than did the birds without the landmarks (closest stop: 44.2 ± 9.1 cm). These distances were not significantly different to those in Experiment 2 (averaged model for closest stop: experiment: *z* = 1.59, *p* = 0.11), suggesting that the additional experience did little to affect how birds stopped around the landmarks. The distance of the closest ‘fly-bys’ by the birds was also not significantly different between Experiments 2 and 3 (averaged model for closest fly-by: experiment: *z* = 1.05, *p* = 0.30), but birds with the landmarks also no longer flew significantly closer to the flower’s location than birds without the landmarks (landmarks: 11.3 ± 2.6 cm; no landmarks: 23.7 ± 7.2 cm; averaged model for closest fly-by: *z* = 0.925, *p* = 0.36, figure 2a). The fly-by accuracy without the landmarks was very similar to those seen in a previous study looking at hummingbird flight paths following removal of a flower [11], albeit one in which hummingbirds were trained without landmarks for 8−12 trials (Experiment 3 fly-by without landmarks: 23.7 ± 7.2 cm; three-dimensional distance for Experiment 2, small flower treatment in [11]: 24.4 cm ± 7.3 cm, estimated from figure 3 in [11]). To look more closely at this discrepancy between the closest stop and the closest fly-by, we calculated the difference between these two measures. As in Experiment 2, the difference between the closest stop and the closest fly-by was very small, barely more than a centimetre when the landmarks were present (1.1 ± 0.6 cm), while the difference between the closest stop and the closest fly-by was almost 20 times higher when the landmarks had been removed (averaged model for difference between stop and fly-by: landmark: 20.4 ± 7.7 cm; *z* = 2.65, *p* = 0.007, figure 2b).

The spacing between stops did not significantly change as the birds gained experience (averaged model for distance between stops: experiment: *z* = 1.56, *p* = 0.24), but we once again found that birds without the landmarks had greater distances between their stops (averaged model for distance between stops: landmark: *z* = 2.17, *p* = 0.03). As in Experiment 2, the overall distribution of distance between stops was largely similar regardless of the presence of landmarks, but this significant difference was driven by a small number of large distances seen only in birds that had the landmarks removed (3/130 distances greater than 2.5 m).

The HMM analyses for Experiment 3 were very similar to those we found in Experiment 2, with no apparent effect of the additional training on hummingbird movements. As in the other experiments, the models identified two movement states corresponding to Travel (faster and less turning) and Search (slower and more turning), and once again, in the best fitting model the probability of switching between these states was determined by an interaction between the bird’s current distance to the flower’s previous location and the presence or absence of the landmarks. When the landmarks were present, the likelihood of switching from Travel to Search increased as birds flew closer to the flower’s former location (electronic supplementary material, figure S2), whereas when the landmarks were removed transition probabilities were the same regardless of distance. As a result, the probability of being in the Search state was tuned to the flower’s previous location only when the landmarks were present (figure 2c). This can be seen in the heatmaps showing where the Search state occurred around the landmarks: birds with the landmarks focusing their searching on the side of the landmarks where the flower had been; birds without the landmarks showed a diffuse search across the wider area (figure 3c,f).

## 4. Discussion

Across these experiments, both traditional comparative cognition methods and movement ecology analyses provided clear evidence that birds remembered the location of the flower, that this memory only requires a single visit to the flower, and that local landmarks were necessary for focusing search around the flower’s previous location. What differs between these measures, however, is the scale over which we see the effects of removing the local landmarks. The comparative cognition measures (stop distances and fly-by distances) focus on how accurate hummingbirds *can* be, finding a 30 cm reduction in stopping accuracy when the landmarks are removed. In contrast, the HMM look along the entire flight path and find that removing the landmarks results in a decoupling of slower Searching movements from the flower’s location. Together these results suggest that birds with the landmarks removed are not simply less accurate than are birds with the landmarks present but may have adopted a fundamentally different search strategy: sampling across a wide area of space rather than using their memory of landmarks to restrict search to a smaller area.

This suggestion that performance in these experiments is not only the result of spatial memory ability but also of the adoption of distinct strategies provides a valuable new direction for studying how wild birds use information during foraging. Our previous studies of hummingbird spatial memory have taken inspiration from laboratory hidden-food paradigms [8,9], in which pigeons *Columbia livia* or Clark’s nutcrackers *Nucifraga columbiana* peck or dig in the location they expect to find food. As birds cannot tell that the food is not present during tests, we consider the first few search locations to be accurate representations of where birds think the reward is located. But the flowers in our studies were not hidden, and so alongside asking *how* hummingbirds stopped so close to the flower’s location, it is also worth asking *why* they stopped so close because we presume they could see that the flower was not there. One possible answer to this question comes from considering why hummingbirds stopped and hovered in the first place. When flying fast, visual blur can make seeing in high resolution difficult [20], and so it seems reasonable to suggest that hovering by searching hummingbirds functions as a means of *information seeking*, and a bird takes the opportunity to observe the world clearly while its head is stationary. To understand how organisms choose where to search next, information seeking in other animals, including humans, has been investigated used frameworks such as Bayes theory or statistical decision theory [21–24]. In studies of human eye movements, for example, search strategy is dictated not only by memory for a stimulus’s location but also by an observer’s confidence in this information. When people are confident in the remembered location, they weight this memory more heavily when deciding where to look next [25], whereas when the observer is not confident then the eye might be drawn to other areas to reduce uncertainty [24,26]. If we consider hummingbird hovering decisions through this lens, we might better understand how these birds use spatial memory to guide search. As in human gaze decisions, the degree to which hummingbirds rely on their prior knowledge might depend on their confidence about this information. When the world appears similar to a hummingbird’s expectation, then it might continue to rely on its prior knowledge. This would result in a memory-driven search process in which the bird looks for information close to the remembered location. If, because landmarks were removed, the array is moved too far between trials [27], or the landmarks become too different to a remembered view [24], spatial cues have changed too much from the bird’s expectation, it might become less confident in this memory and so rely more on a systematic search focused on seeking information across a wider area. Although hummingbirds strongly prefer to use spatial information over other cues when they return to a rewarded location [28,29], during a systematic search, hovering decisions might rely less on spatial knowledge and focus more on the potential to gain information. As a result, even if a hummingbird accurately knows the location of the flower, this accuracy might not be reflected in where he chooses to hover if the bird is uncertain about the state of the world. This would explain why, for example, the difference in accuracy between birds with and without the landmarks was much more pronounced in stops than in fly-bys, and the difference between stops and fly-bys was much larger when the landmarks were removed (figure 2b). Fly-bys might, then, be a better measure of how close hummingbird *can* get to a flower’s location, with stops representing only what birds can do when their confidence is high. By reframing hummingbird search as information seeking rather than as a read-out of spatial memory, we can better examine how the behaviour we observe relates to what birds might know.

This holistic view of hummingbird search behaviour is enabled because we analysed changes in behaviour along the flight path. While changes in movement paths have been used to probe spatial cognition in the past, notably the ‘change-point’ analysis used with primates [30], the benefit of HMMs and other movement ecology models comes from their ability to analyse the impact of multiple different factors on these decisions, making them well suited to behavioural experiments. Here, we could model the likelihood of searching based on the distance of the bird to the goal and the experimental treatment, but one might also model changes in probabilities as animals learn or based on distance to different predicted locations or even as estimated sensory cues change, such as the apparent size of a landmark [31]. This hybrid modelling approach differs from the experimental approaches used in the majority of comparative cognition studies but reflects a growing trend in behavioural neuroscience and neuroethology of combining high-resolution tracking with computational modelling approaches [32–34]. While there is a long history of using modelling to investigate cognitive processes in animals [35], these models have tended to be analytical or computational rather than purely statistical in nature, and compared with data only in terms of how well they predict which of a small set of behaviours animals may perform under different circumstances (e.g [36]). Movement ecology tools, in contrast, are designed to explain movement and so are used to analyse high-resolution behavioural recordings. There is also considerable interest within movement ecology for understanding the impact of cognitive processes on animal movement [4,5,37], a topic ideally suited for collaboration with comparative cognition researchers. We believe it would be mutually beneficial for comparative cognition researchers to familiarize themselves with the work being carried out by movement ecologists and with the tools that are being developed in that field.

By combining movement ecology tools with experimental methods, our study also overcomes a central challenge facing movement ecologists: interpreting what a behavioural state ‘means’. HMMs have become very popular for segmenting paths into different potential movement states, but what these states actually mean in biological terms can be very hard to determine. Although ‘Search’ and ‘Foraging’ states may overlap with other indicators of foraging, such as diving in seabirds [38], the motivation of these animals is ultimately unknowable. In our study, however, the motivation of the hummingbirds was clear. Birds flew from a perch to a location where they had found a food source and, upon finding it missing, searched the surrounding area. While the states identified by the HMMs, Travel and Search, resemble states that have been identified in many movement ecology studies [38], we can be much more confident that the Search state in our study truly reflects a searching process by the birds. Although our study occurred over a smaller scale, and over shorter periods of time, than most studies in movement ecology, we would maintain that this is balanced by a better understanding of the contexts in which the behaviour occurred. This balance between the scale of movement and the interpretability of the data probably reflects a fundamental trade-off for researchers interested in animal movement, but one that has been highly skewed towards studying animals at larger scales. Just as a formal collaboration between comparative cognition offers opportunities to cognition researchers looking to apply modelling approaches to study behaviour, the establishment of small-scale to medium-scale experimental studies using movement ecology tools with freely moving animals could be used to ground-truth models and patterns of behaviour resulting in these models being more interpretable at much larger scales.

Movement ecology and comparative cognition have largely existed in separate intellectual spaces, with tools developed for studying the long-distance tracked paths of wild animals rarely applied to any other purpose. In our interdisciplinary study, we combined comparative cognition experiments with movement ecology models and gained a new perspective on hummingbird decision-making. Rather than simply ‘searching further away’ when familiar landmarks were removed, we discovered that hummingbirds changed their behaviour along the entire flight path, suggesting that birds switched from a memory-based search strategy to a more systematic search across a wider area. This study highlights what movement ecology and comparative cognition can offer one another, and we hope initiates a period of increased collaboration between these two fields.

## Data Availability

The data and code for analysis of distances can be found at [39], while the code for the HMMs can be found at [40]. Supplementary material is available online [41].
